# Bloodstream infections caused by *Klebsiella pneumoniae*: prevalence of *bla*_KPC_, virulence factors and their impacts on clinical outcome

**DOI:** 10.1186/s12879-018-3263-x

**Published:** 2018-07-31

**Authors:** Min Xu, Yiqi Fu, Haishen Kong, Xiao Chen, Yu Chen, Lanjuan Li, Qing Yang

**Affiliations:** 1 Key Laboratory of Clinical In Vitro Diagnostic Techniques of Zhejiang Province, Center of Clinical Laboratory, The First Affiliated Hospital, College of Medicine, Zhejiang University, Hangzhou, China; 2 Department of Respiratory Diseases, The First Affiliated Hospital, College of Medicine, Zhejiang University, Hangzhou, China; 3 State Key Laboratory for Diagnosis and Treatment of Infectious Diseases, Collaborative Innovation Center for Diagnosis and Treatment of Infectious Diseases, The First Affiliated Hospital, College of Medicine, Zhejiang University, Hangzhou, China

**Keywords:** Bloodstream infections, *Klebsiella pneumoniae*, KPC, Hypermucoviscous, Prognosis

## Abstract

**Background:**

*Klebsiella pneumoniae* bloodstream infections (BSIs) occur with significant prevalence and high mortality worldwide. Antimicrobial resistance and virulence are two main factors participating in the pathogenicity of *K. pneumoniae*. Here we investigated the prevalence of *bla*_KPC_ and virulence factors in *K. pneumoniae* isolated from patients with BSIs and their association with clinical outcome.

**Methods:**

The clinical data of 285 *K. pneumoniae* BSI cases diagnosed from January 2013 to December 2015 in a Chinese university hospital were retrospectively evaluated. The “string test” was performed to identify hypermucoviscous *K. pneumoniae* (HMKP). *bla*_KPC_, *rmpA*, *magA* and serotype-specific genes were detected by PCR amplification. Finally, a Cox proportional hazards model was employed to determine the predictors of 14-day mortality.

**Results:**

Of these isolates, the prevalence of *bla*_KPC_ and *rmpA* were 33.3% (95/285) and 31.6% (90/285) respectively. 69 isolates (24.2%, 69/285) were HMKP. *rmpA* was strongly associated with HM phenotype. The KPC-producing KP and HMKP were almost non-overlapping and only three HMKP isolates harbored *bla*_KPC_. K1 (28, 40.6%) and K2 (22, 31.9%) were the most common serotypes in HMKP. 44.9% of HMKP BSIs had origin of biliary tract infection or liver abscess. The 14-day mortality was 100% in *bla*_KPC_^+^/HM^+^ subgroup (3/3), followed by *bla*_KPC_^+^/HM^−^ (39/92, 42.4%), *bla*_KPC_^−^/HM^+^ (5/66, 7.6%) and *bla*_KPC_^−^/HM^−^ (7/124, 5.6%). The 14-day cumulative survival was significantly different between *bla*_KPC_^+^ and *bla*_KPC_^−^ subgroup (Log-rank *p* < 0.001) but almost equal between *bla*_KPC_^−^/HM^+^ and *bla*_KPC_^−^/HM^−^ subgroup (Log-rank *p* = 0.578) under the condition of comparable illness severity between *bla*_KPC_^−^/HM^+^ and *bla*_KPC_^−^/HM^−^ subgroup. Independent risk factors for 14-day mortality were Pitt bacteremia score (HR 1.24, CI 95% 1.13–1.36, *p* < 0.001), Charlson comorbidity index (HR 1.24, CI 95% 1.09–1.41, *p* = 0.001), septic shock (HR 2.61, CI 95% 1.28–5.35, *p* = 0.009) and *bla*_KPC_ (HR 2.20, CI 95% 1.06–4.54, *p* = 0.034).

**Conclusions:**

Most of HMKP were antibiotic-susceptible and people infected received appropriate antimicrobial therapy, which may explain the favorable outcome of HMKP BSIs. The KPC-producing HMKP BSIs were scarce but life-threatening. *bla*_KPC_ was valuable in predicting 14-day mortality.

**Electronic supplementary material:**

The online version of this article (10.1186/s12879-018-3263-x) contains supplementary material, which is available to authorized users.

## Background

*Klebsiella pneumoniae* is the second most common pathogen in Enterobacteriaceae bloodstream infections (BSIs). The reported mortality rate of *K. pneumoniae* BSIs varies from 15 to 79% [1–4], which was lower than that of *Acinetobacter baumannii* BSIs (30 to 84%) [5, 6] but higher than *Escherichia coli* (5 to 22%) [7–9]. Antimicrobial resistance and virulence are generally considered as significant factors in the pathogenicity of *K. pneumoniae*. During the past years, as the increasing of antimicrobial resistance, especially the emerging of carbapenem-resistant *K. pneumoniae*, a serious dilemma has been posed to clinical therapy [10]. *K. pneumoniae* carbapenemase (KPC) is the most important genetic mechanism of carbapenem resistance [11]. Several studies that focus on KPC-producing *K. pneumoniae* (KPC-KP) BSI have found a high mortality rate [2–4]. However, similar large research in our geographical area is still missing.

Hypervirulent *K. pneumoniae* (hvKP), traditionally characterized as hypermucoviscous (HM) phenotype, has emerged as a significant pathogen since first described in 1986 [12]. The HM phenotype has been proved to be strongly associated with *rmpA* (regulator of the mucoid phenotype gene *A*) [13]. The other virulence-associated factors in hvKP include *magA* (mucoviscosity-associated gene *A*) and capsular polysaccharide, especially K1 and K2 serotypes. These factors confer *K. pneumoniae* the ability to colonize mucous surface and evade the phagocytosis of immune cells [14]. hvKP is usually associated with severe infectious diseases, such as pyogenic liver abscess, endophthalmitis, meningitis, and necrotizing fasciitis [15, 16], but only limited information is available about hvKP BSIs [1, 17, 18]. Studies in animal model have shown that both the HM phenotype and the capsular serotypes (K1 and K2) are strongly associated with death [19], but only small amounts of clinical studies with small sample size have evaluated the impact of these virulence factors in the outcome of *K. pneumoniae* BSIs hitherto, and the results are still controversial [1, 18, 20].

Generally, most HMKP are only resistant to ampicillin. There is largely non-overlapping between antimicrobial-resistant and HM phenotype, but recent study shows the antimicrobial resistant rate of HMKP is increasing [20]. Some cases of KPC-producing HMKP infections have been already reported in China [21, 22]. More surveillance is needed to illustrate the prevalence of KPC-producing HMKP strains. Therefore, we conducted this three-year period study of *K. pneumoniae* BSIs in a Chinese university hospital for two main objects: investigate microbial characteristics including the prevalence of *bla*_KPC_ responsible for carbapenem resistance, and several virulence-associated factors; and analyze their impact on clinical outcomes.

## Methods

### Setting and design

This retrospective cohort study was conducted in The First Affiliated Hospital, College of Medicine, Zhejiang University, Hangzhou, China, a 2500-bed tertiary hospital having approximately 131,000 admissions each year, and three intensive care units (ICU): a 29-bed medical ICU, a 29-bed surgical ICU and a 15-bed emergency ICU. There is infectious diseases service at this hospital.

The patients aged over 18 years and developed *K. pneumoniae* BSIs during hospitalization were identified between January 2013 and December 2015. Only the first episode for each patient was included in our analysis. Patients with polymicrobial bacteremia were excluded. The relevant clinical and microbiological data we needed were extracted from the electronic or paper medical records and microbiologic database. In present study, the data was de-identified and hence informed consent was waived. Ethical approval was granted from the Ethics Committees and review board of the First Affiliated Hospital, College of Medicine, Zhejiang University.

### Variables and definitions

A *K. pneumoniae* BSIs were defined as an infection confirmed by blood culture positive for *K. pneumoniae* and clinical signs of the systemic inflammatory response syndrome [23].

The following data were collected: demographics (sex, age), date and unit of hospitalization; infections acquisition categorized as either nosocomial or community-acquired [20]; baseline severity of illness estimated by the Acute Physiology and Chronic Health Evaluation (APACHE) II score; comorbidities and severity of underlying diseases assessed by the Charlson comorbidity index [24]; severity of bacteremia calculated using Pitt bacteremia score [25]; and source of bacteremia. Immunosuppressive state (chemotherapy, radiotherapy and/or immunosuppressive drugs), septic shock and multiple organ dysfunction syndrome (MODS) at the onset of BSI were also documented. Empirical antibiotics therapy was defined as therapy given within 24 h of the culture being taken, while definitive antimicrobial therapy was considered the administration of antibiotics for at least 48 h. Antibiotics therapy was considered appropriate if it include at least on antimicrobial agent to which the causative pathogen displayed in vitro susceptibility [26].

### Assessment of the resistance profile and *bla*_KPC_

The isolate identification and antimicrobial susceptibility testing were conducted by Vitek 2 automated system (bioMérieux, France). Minimum inhibitory concentrations (MICs) were categories according to the breakpoints defined by Clinical and Laboratory Standards Institute (CLSI, 2016). For tigecycline, disk diffusion method was used and the results were interpreted according to the U. S. Food and Drug Administration criteria (susceptible ≥19 mm, resistant < 14 mm).

The presence of the *bla*_KPC_ was investigated by PCR and sequencing [27].

### Detection of the HM phenotype, capsular serotype and virulence genes

The “string test” was used to identify HM phenotype. A positive string test was defined as the generation of a viscous string of > 5 mm in length, when using a bacteriology loop to stretch bacterial colony cultured on an agar plate overnight at 37 °C. [14].

The virulence genes *magA*, *rmpA*, and serotype-specific genes for the K1, K2, K5, K20, K54 and K57 capsular serotype were amplified by PCR as described previously [20].

### Multilocus sequence typing (MLST)

In order to identify prevalent high-risk clones, MLST of the isolates from the non-survivors subgroup was performed. It was done with seven housekeeping genes (*gapA*, *infB*, *mdh*, *phoE*, *pgi*, *rpoB* and *tonB*) according to the protocol on the MLST website [28]. Sequence types (STs) that had not been described previously were submitted to the database.

### Statistical analysis

Continuous variables were expressed as mean and standard deviation (SD) for normally distributed data, or as median and interquartile rang (IQR) for non-normal data. Categorical variables were presented as absolute numbers and their relative frequencies. The two-tailed t test or Mann-Whitney test was used for continuous variables, and the chi-square test or Fisher exact test was used for categorical variables, when appropriate. A Cox regression model by including the variables emerging from univariate analysis with *P* value < 0.1 as well as clinically important were applied to analysis the effect of different variables on 14-day mortality. In order to estimate the risk of death, survival curves were constructed with the Kaplan-Meier method and log-rank test. All the statistical analyses were two-tailed, and *P* value ≤0.05 was considered significant. The SPSS software (version 20.0) was used for data analysis in the present study.

## Results

### Microbiological characteristics

During the study period, a total of 285 *K. pneumoniae* BSI cases that met the inclusion criteria were identified. The characteristics of antimicrobial resistance and virulence-associated factors according to *bla*_KPC_ and HM phenotype are shown in Table 1.

**Table 1:** *bla*_KPC_ and virulence-associated factors of the 285 *K. pneumoniae* isolated from BSIs

Characteristics	Total (*n* = 285)	*bla*_KPC_^+^	*bla*_KPC_^−^	*P* value^a^	HMKP	cKP	*P* value^b^
		(*n* = 95)	(*n* = 190)		(*n* = 69)	(*n* = 216)	
*bla*_KPC_	95 (33.3)	–	–	–	3 (4.3)	92 (42.6)	< 0.001
Virulence factors							
HM phenotype	69 (24.2)	3 (3.2)	66 (34.7)	< 0.001	–	–	–
K1	28 (9.8)	1 (1.1)	27 (14.2)	< 0.001	18 (26.1)	10 (4.6)	< 0.001
K2	22 (7.7)	0	22 (11.6)	0.001	21 (30.4)	1 (0.5)	< 0.001
K5	3 (1.1)	0	3 (1.6)	0.55	2 (2.9)	1 (0.5)	0.15
K20	3 (1.1)	1 (1.1)	2 (1.1)	0.99	2 (2.9)	1 (0.5)	0.15
K54	6 (2.1)	0	6 (3.2)	0.18	4 (5.8)	2 (0.9)	0.03
K57	3 (1.1)	0	3 (1.6)	0.55	3 (4.3)	N	0.01
K-nontypable	220 (77.2)	93 (97.9)	127 (66.8)	< 0.001	19 (27.5)	201 (93.1)	< 0.001
*magA*	24 (8.4)	0	24 (12.6)	< 0.001	17 (24.6)	7 (3.2)	< 0.001
*rmpA*	90 (31.6)	2 (2.1)	88 (46.3)	< 0.001	62 (89.9)	28 (13.0)	< 0.001

Data are presented as No. (%) unless otherwise specified

*Abbreviations*: *HM* hypermucoviscous, *HMKP* hypermucoviscous *K. pneumoniae*, *cKP* classic *K. pneumoniae*

^a^*bla*_KPC_^+^ vs *bla*_KPC_^−^

^b^HMKP vs cKP

34.7% isolates (99/285) displayed non-susceptibility to carbapenem, most of which (92.9%, 92/99) carried *bla*_KPC_. There were another three *bla*_KPC_ positive isolates were carbapenem susceptible. Amikacin and tigecycline had the lowest non-susceptible rates among the studied drugs. The detailed antimicrobial non-susceptibility profiles of the 15 drugs are listed in Additional file 1: Table S1.

The HM phenotype was found in 69 (24.2%) isolates. K1 (28 isolates) and K2 (22 isolates) were the most common serotypes. PCR detected virulence gene *rmpA* in 90 isolates (31.6%) and *magA* in 24 isolates (8.4%). The detection rates of K1, K2, *rmpA* and *magA* were significantly higher in HM^+^ group than HM^−^ group. Multivariate analysis showed *rmpA* and K2 to be the independent factor truly associated with HM phenotype (Additional file 2: Table S2).

Compared with non-KPC-KP, KPC-KP showed much more antimicrobial resistance, but less HM phenotype, capsular serotype, *magA* and *rmpA*.

The HMKP and KPC-producing KP were almost non-overlapping. Three HMKP were proved to carry *bla*_KPC_, in which two isolates also harbored capsular serotype genes and *rmpA*.

### Clinical characteristics according to *bla*_KPC_ and HM phenotype

According to *bla*_KPC_ and HM phenotype, the 285 KP BSI cases were divided into four subgroups, *bla*_KPC_^+^/HM^+^, *bla*_KPC_^+^/HM^−^, *bla*_KPC_^−^/HM^+^, and *bla*_KPC_^−^/HM^−^. The baseline demographic and clinical characteristics are shown in Table 2.

**Table 2:** The clinical characteristics of *K. pneumoniae* BSIs according to *bla*_KPC_ and HM phenotype

Characteristics	*bla*_KPC_^+^/HM^+^ (n = 3)	*bla*_KPC_^+^/HM^−^ (*n* = 92)	*bla*_KPC_^−^/HM^+^ (*n* = 66)	*bla*_KPC_^−^/HM^−^ (*n* = 124)	*P* value^c^	*P* value^d^	*P* value^e^
Demographic data							
Age (years), mean (±SD)	68.0 ± 19.2	57.4 ± 15.3	57.1 ± 13.9	56.0 ± 17.4	0.93	0.94	0.88
Male sex	2 (66.7)	68 (73.9)	44 (66.7)	85 (68.5)	0.32	0.39	0.79
Hospitalization unit							
Medical	0	55 (59.8)	56 (84.8)	105 (84.7)	**0.001**	**< 0.001**	0.98
ICU	3 (100)	37 (40.2)	10 (15.2)	19 (15.3)			
Acquisition							
Nosocomial	2 (66.7)	89 (96.7)	29 (43.9)	86 (69.4)	**< 0.001**	**< 0.001**	**0.001**
Community	1 (33.3)	3 (3.3)	37 (56.1)	38 (30.6)			
Underlying diseases							
Diabetes	0	22 (23.9)	11 (16.7)	30 (24.2)	0.27	0.96	0.23
Hematologic malignancy	1 (33.3)	7 (7.6)	3 (4.5)	20 (16.1)	0.52	0.06	**0.02**
Solid tumor	1 (33.3)	11 (12.0)	21 (31.8)	34 (27.4)	**0.002**	**0.006**	0.52
Solid organ transplantation	0	21 (22.8)	4 (6.1)	8 (6.5)	**0.004**	**< 0.001**	0.92
Heart disease	0	16 (17.4)	8 (12.1)	11 (8.9)	0.36	0.06	0.48
Chronic lung disease	0	10 (10.9)	10 (15.2)	8 (6.5)	0.43	0.25	0.051
Chronic kidney disease	1 (33.3)	31 (33.7)	8 (12.1)	22 (17.7)	**0.002**	**0.007**	0.31
Origin of BSI							
Respiratory tract	2 (66.7)	29 (31.5)	7 (10.6)	11 (8.9)	**0.002**	**0.001**	0.14
Intraabdominal	1 (33.3)	19 (20.7)	9 (13.6)	7 (5.6)	0.26	**0.001**	0.06
Soft tissue	0	4 (4.3)	4 (6.1)	2 (1.6)	0.72	0.41	0.19
Intravenous catheter	0	6 (6.5)	0	4 (3.2)	**0.04**	0.42	0.30
Urinary tract	0	4 (4.3)	1 (1.5)	9 (7.3)	0.40	0.37	0.18
Biliary tract	0	5 (5.4)	16 (24.2)	31 (25)	**0.001**	**< 0.001**	0.91
Liver abscess	0	3 (3.3)	15 (22.7)	10 (8.1)	**< 0.001**	0.14	**0.004**
Unknown	0	14 (15.2)	12 (18.2)	46 (37.1)	0.62	**< 0.001**	**0.007**
Other(s)^a^	0	8 (8.7)	2 (3.0)	3 (2.4)	0.20	0.08	0.80
Clinical presentation							
APACHE II score, median (IQR)	25 (23.5–26.5)	23 (18–30)	14 (11–20)	15 (11–20)	**< 0.001**	**< 0.001**	0.96
Pitt bacteremia score, median (IQR)	4 (3–4.5)	7 (2–9)	1 (1–2)	1 (0–2)	**< 0.001**	**< 0.001**	0.60
Charlson comorbidity index, median (IQR)	2^b^	3 (1–4)	2 (1–3)	2 (1–4)	0.56	0.40	0.81
Septic shock	2 (66.7)	46 (50.0)	10 (15.2)	11 (8.9)	**< 0.001**	**< 0.001**	0.19
MODS	2 (66.7)	37 (40.2)	2 (3.0)	7 (5.6)	**< 0.001**	**< 0.001**	0.65
Immunosuppression state	1 (33.3)	45 (48.9)	8 (12.1)	40 (32.3)	**< 0.001**	**0.013**	**0.002**
Therapy							
Appropriate empiric therapy	0	33 (35.9)	60 (90.9)	96 (77.4)	**< 0.001**	**< 0.001**	**0.02**
Appropriate definitive therapy	0	60 (65.2)	65 (98.5)	105 (84.7)	**< 0.001**	**0.001**	**0.003**
14-day mortality	3 (100)	39 (42.4)	5 (7.6)	7 (5.6)	**< 0.001**	**< 0.001**	0.84

Data are presented as No. (%) unless otherwise specified; boldface, indicates statistical significance (*p*<0.05)

*Abbreviations*: *HM* hypermucoviscous, *BSIs* bloodstream infections, *CNS* central nervous system, *APACHE* Acute Physiology and Chronic Health Evaluation, *MODS* multiple organ dysfunction syndrome, *SD* standard deviation, *IQR* interquartile range

^a^Central nervous system infection, 9 cases; mediastinal infection, 1 cases; endocarditis, 3 cases

^b^The three cases had same Charlson comorbidity index

^c^*bla*_KPC_^+^/HM^−^ vs *bla*_KPC_^−^/HM^+^

^d^*bla*_KPC_^+^/HM^−^ vs *bla*_KPC_^−^/HM^−^

^e^*bla*_KPC_^−^/HM^+^ vs *bla*_KPC_^−^/HM^−^

Due to the limited number, *bla*_KPC_^+^/HM^+^ subgroup was not included in statistical analysis.

To compare with *bla*_KPC_^−^/HM^+^ and *bla*_KPC_^+^/HM^−^ subgroups, *bla*_KPC_^+^/HM^−^ subgroup showed apparently distinct characterization by statistical analysis. Patients in the latter subgroup were more likely to be nosocomial acquired infection (96.7%) and have ICU hospitalization (40.2%). Respiratory tract source of BSIs was more frequent in *bla*_KPC_^+^/HM^−^ subgroup, but fewer biliary tract or liver abscess origin. This group also had much severer illness process (higher APACHE II score and Pitt bacteremia score; more incidence of MODS and septic shock), less opportunity to receive appropriate antibiotic therapy and much higher 14-day mortality.

There were no significant differences in demographic data, underlying diseases, Charlson comorbidity index, severity of illness evaluation (APACHE II score, Pitt bacteremia score, MODS and septic shock) and 14-day mortality between the *bla*_KPC_^−^/HM^+^ and *bla*_KPC_^−^/HM^−^ subgroups. However, community acquired infection, liver abscess source of BSIs and immunocompetent state were more frequent in *bla*_KPC_^−^/HM^+^ subgroup.

### Outcomes and mortality predictors

Most fatalities occurred within 14 days after *K. pneumoniae* BSIs onset (18.9%, 54/285) and the in-hospital mortality rate was 32.6% (93/285). The 14-day mortality of three *bla*_KPC_^+^/HM^+^ KP-BSI cases was 100%, followed by *bla*_KPC_^+^/HM^−^ (42.4%), *bla*_KPC_^−^/HM^+^ (7.6%) and *bla*_KPC_^−^/HM^−^ subgroup (5.6%).

**Fig. 1:**
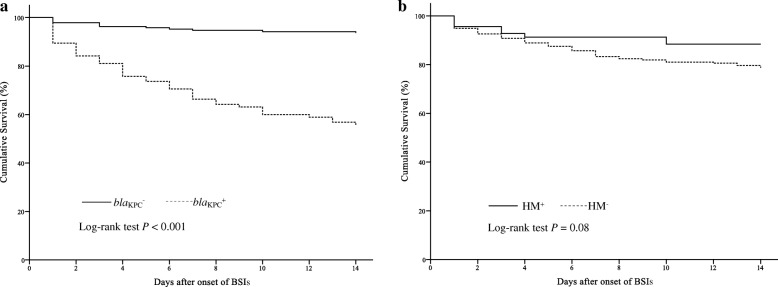
Kaplan-Meier curves about impact of *bla*_KPC_ (**a**) and HM phenotype (**b**) on 14-day survival. HM, hypermucoviscous; BSIs, bloodstream infections

Kaplan-Meier curves were used to evaluate the impact of *bla*_KPC_ and HM phenotype on 14-day survival. As shown in Fig. 1a, the 14-day cumulative survival was significantly different between *bla*_KPC_^+^ and *bla*_KPC_^−^ group (55.8% versus 93.7%, *P* < 0.001). Although the 14-day cumulative survival of HM^−^ group (78.7%) was lower than that of HM^+^ group (88.4%), the difference was not statistically significant (*P* = 0.08, Fig. 1b). The prevalence of *bla*_KPC_ was significantly higher in HM^−^ group than in HM^+^ group (42.6% versus 4.3%, *P* < 0.001. Table 1), which may contribute to the relatively low survival in HM^−^ group. In order to exclude the influence of *bla*_KPC_, comparison of the 14-day cumulative survival was performed between *bla*_KPC_^−^/HM^+^ and *bla*_KPC_^−^/HM^−^ subgroups. It also showed minor impact of HM phenotype on outcome (cumulative survival, 92.4% for *bla*_KPC_^−^/HM^+^ versus 94.4% for *bla*_KPC_^−^/HM^−^; Log-rank *P* = 0.578) under the condition of comparable illness severity between *bla*_KPC_^−^/HM^+^ and *bla*_KPC_^−^/HM^−^ subgroups.

In the univariate analysis, several clinical and microbial factors were significantly associated with mortality (Table 3). Patients in nonsurvivor group were more likely to have ICU hospitalization, nosocomial acquired infection, chronic kidney disease, heart disease, and immunosuppression state. This group also had severer illness evaluation and higher prevalence of *bla*_KPC_. However, biliary tract or liver abscess sourced BSI, appropriate empiric or definitive antibiotic therapy, *rmpA* positive and HM phenotype were associated with survival.

**Table 3:** Univariate and multivariate analysis of factors associated with 14-day mortality

Characteristic	Total	Survivors	Non-survivors	*P* value	Multivariate analysis	
	(n = 285)	(*n* = 231)	(*n* = 54)		HR (95% CI)	*P* value
Demographic data						
Age (years), mean (±SD)	56.8 ± 15.9	56.7 ± 16.4	57.2 ± 14.1	0.83		
Male sex	199 (69.8)	159 (68.8)	40 (74.1)	0.45		
ICU hospitalization	69 (24.2)	48 (20.8)	21 (38.9)	**0.005**		
Nosocomial acquisition	206 (72.3)	158 (68.4)	48 (88.9)	**0.002**		
Underlying diseases						
Diabetes	63 (22.1)	52 (22.5)	11 (20.4)	0.73		
Hematologic malignancy	31 (10.9)	23 (10.0)	8 (14.8)	0.30		
Solid tumor	67 (23.5)	60 (26.0)	7 (13.0)	**0.04**		
Solid organ transplantation	33 (11.6)	24 (10.4)	9 (16.7)	0.19		
Heart disease	35 (12.3)	23 (10.0)	12 (22.2)	**0.01**		
Chronic lung disease	28 (9.8)	25 (10.8)	3 (5.6)	0.24		
Chronic kidney disease	62 (21.8)	38 (16.5)	24 (44.4)	**< 0.001**		
Origin of BSI						
Respiratory tract	49 (17.2)	28 (12.1)	21 (38.9)	**< 0.001**		
Intraabdominal	36 (12.6)	26 (11.3)	10 (18.5)	0.15		
Soft tissue	10 (3.5)	6 (2.6)	4 (7.4)	0.19		
Intravenous catheter	10 (3.5)	9 (3.9)	1 (1.9)	0.75		
Urinary tract	14 (4.9)	14 (6.1)	0	0.13		
Biliary tract	52 (18.2)	51 (22.1)	1 (1.9)	**0.001**		
Liver abscess	28 (9.8)	28 (12.1)	0	**0.007**		
Unknown	72 (25.3)	60 (26.0)	12 (22.2)	0.57		
Other(s)	13 (4.6)	8 (3.5)	5 (9.3)	0.14		
Clinical presentation						
APACHE II score, median (IQR)	18 (12–24)	15 (11–21)	26 (21–33.3)	**< 0.001**		
Pitt bacteremia score, median (IQR)	2 (1–6)	1 (0–3)	8 (4.75–10.5)	**< 0.001**	1.24 (1.13–1.36)	**< 0.001**
Charlson comorbidity index, median (IQR)	2 (1–4)	2 (1–4)	3 (2–5)	**0.02**	1.24 (1.09–1.41)	**0.001**
Septic shock	69 (24.2)	30 (13.0)	39 (72.2)	**< 0.001**	2.61 (1.28–5.35)	**0.009**
MODS	48 (16.8)	19 (8.2)	29 (53.7)	**< 0.001**		
Immunosuppression state	94 (33.0)	67 (29.0)	27 (50.0)	**0.003**		
Therapy						
Appropriate empiric therapy	189 (66.3)	163 (70.6)	26 (48.1)	**0.002**		
Appropriate definitive therapy	230 (80.7)	194 (84.0)	36 (66.7)	**0.004**		
HM phenotype	69 (24.2)	61 (26.4)	8 (14.8)	0.08		
*bla*_KPC_	95 (33.3)	53 (22.9)	42 (77.8)	**< 0.001**	2.20 (1.06–4.54)	**0.034**
*rmpA*	90 (31.6)	83 (35.9)	7 (13.0)	**0.001**		

Data are presented as No. (%) unless otherwise specified; boldface, indicates statistical significance (*p*<0.05)

*Abbreviations*: *HM* hypermucoviscous, *BSIs* bloodstream infection, *CNS* central nervous system, *APACHE* Acute Physiology and Chronic Health Evaluation, *MODS* multiple organ dysfunction syndrome, *SD* standard deviation, *IQR* interquartile range, *CI* confidence interval, *HR* hazard ratio

Cox regression analysis identified Charlson comorbidity index (HR 1.24, CI 95% 1.09–1.41, *P* = 0.001), septic shock (HR 2.61, CI 95% 1.28–5.35, *P* = 0.009), Pitt bacteremia score (HR 1.24, CI 95% 1.13–1.36, *P* < 0.001), and *bla*_KPC_ (HR 2.20, CI 95% 1.06–4.54, *P* = 0.034) as independent predictors of 14-day crude mortality (Table 3).

### Molecular genotyping

MLST of the 54 isolates from the 14-day nonsurvival subgroup identified 19 sequence types (STs), as shown in Additional file 3: Figure S1. ST11 was the most prevalent (*n* = 36, 66.7%). The other STs had only one isolate each. All of the ST11 isolates were *bla*_KPC_ positive except one isolate. Two cases caused by ST11 were defined as community-acquired infection. However, both of the patients had been hospitalized during previous 2 months. The remaining 34 ST11 cases were unambiguous nosocomial infection. Looking at the detailed clinical epidemiological information of all the 36 ST11 cases, there was no sudden increase in incidence of the infection during this period and the patients who had a close sampling time had non-overlapping stays in the same unit, suggesting just endemicity of ST11 clone in our hospital and nil outbreak happened. Eight HMKP isolates had unique STs. Three *bla*_KPC_^+^/HM^+^ isolates were ST11, ST893 and ST15, and had apparent diversity in genetic background.

## Discussion

The retrospective study involved 285 *K. pneumoniae* BSI cases occurred from January 2013 to December 2015 in a Chinese hospital. The prevalence of *bla*_KPC_ and several virulence-associated factors were investigated, as well as the impact of these factors on mortality. It represented one of the largest investigations of *K. pneumoniae* BSIs in Chinese hospital that focused on microbial and clinical characterization simultaneously.

In this study, the carbapenem resistant rate of *K. pneumoniae* isolated from BSIs was 34.7%, which was much higher than the result of a national investigation reported by Xu et al. in China (5.5%) [29]. The disparity can be attributed to the variant distribution of *bla*_KPC_ among regions. As the most important carbapenemase gene of *K. pneumoniae*, *bla*_KPC_ was detected in 33.3% of 285 isolates. Although there was no information concerning the detection frequency of *bla*_KPC_ in *K. pneumoniae* isolated from BSIs in China hereto, this data was higher than that reported by Yedidah et al. (25.0%) in Israel [3]. MLST showed 66.7% of isolates from the 14-day nonsurvival group was ST11, which belonged to CC258, a well-known international high-risk MDR clone of *K. pneumoniae* [30]. Almost all of the ST11 isolates were *bla*_KPC_ positive and caused nosocomial infection in this study. It is believed that the nosocomial clonal dissemination of KPC-producing ST11 *K. pneumoniae* played a significant role in the high detection rate of *bla*_KPC_. In addition, the positive rate of *bla*_KPC_ was 44.2% in isolates from nosocomial-acquired infection, but only 5.1% in isolates from community-acquired infection. Therefore, including of more nosocomial infection cases (72.3%) and relatively less community infection cases (27.7%) may also lead to the increased overall carbapenem resistant rate.

The study showed 24.2% (69/285) of *K. pneumoniae* BSI cases were caused by HMKP. This percentage was much higher than that recently reported by Cubero et al. in Spain (5.4%) [17], but lower than that described in other regions of China (31.4% [1] and 28% [20]). In accordance with a number of previous studies, K1 and K2 were the most common serotypes [1, 17, 20, 31]. However, the prevalence of these two serotypes in this study was lower than those reported by other Chinese researchers [1, 20]. Besides the geographic variation, the discrepancy in sample size might account for these differences.

As expected, most HMKP displayed susceptibility to carbapenem (94.2%, 65/69), which was consistent to the traditional view that HMKP is rarely resistant to antibiotics except its intrinsic resistance to ampicillin [21]. However, recent studies have indicated that the degree of antimicrobial resistance of HMKP strains increased over time [20]. Even KPC-producing HMKP isolates have been reported in China [22, 32]. In our study, 4.3% (3 isolates) isolates were identified as KPC-producing HMKP strains and they belonged to three distinctive STs (ST11, ST15 and ST893) without molecular epidemiological relationship, suggesting they were sporadic cases. The isolates of ST11 and ST15 were K-nontypable and *rmpA* and *magA* negative. Other factors may be responsible for the HM phenotype. In addition, as far as our best knowledge, it is the first report of KPC-producing HMKP ST893 co-harboring K20 serotype and *rmpA*.

When classified according to HM and *bla*_KPC_ phenotype, clinical characteristics were significantly different between subgroups. Besides community acquisition infections (*P* = 0.001), liver abscess (*P* = 0.004) and immune status (*P* = 0.002) were most significantly associated with HMKP (*bla*_KPC_^−^/HM^+^ versus *bla*_KPC_^−^/HM^−^). This feature is consistent with previous reports that HMKP usually infects immunocompetent subjects and causes liver abscess [14]. On the contrary, KPC-KP were more likely to be nosocomial acquisition, happen in patients with serious underlying diseases (solid organ transplantation, central nervous diseases and chronic kidney disease) and have respiratory tract or intraabdominal source. These differences indicate that HMKP and KPC-KP each has unique potential reservoir and pathogenicity.

Several studies have reported the mortality of HMKP BSIs was lower than that of cKP BSIs [1, 31]. Although without significant difference, the trend was also observed in this study (14-day mortality, HMKP 11.6% versus cKP 21.3%, Log-rank *P* = 0.08). Because of low resistant rates, the empirical and definitive anti-infective therapy against HMKP BSIs could be reasonable and effective, which may explain the relatively favorable prognosis. We also performed 14-day survival analysis between *bla*_KPC_^−^/HM^+^ and *bla*_KPC_^−^/HM^−^ subgroups to exclude the interference of resistance. The result also showed HM phenotype alone had minor impact on the poor prognosis of BSIs (14-day mortality, 7.6% versus 5.6%, *P* = 0.578). However, new evidence has suggested that a HM phenotype only is not sufficient to indicate a hypervirulent state [33]. Other important virulence-associated factors such as siderophores were not performed in this study may lead to bias on identification of real hypervirulent *K. pneumoniae*.

Previous studies on KPC-KP BSIs reported mortality rates up to 79% [2]. In this study, the 14-day mortality of KPC-KP BSIs was 44.2%. Compared with patients of non-KPC-KP BSIs, patients of KPC-KP BSIs were more critical (higher APACHE II score and Pitt bacteremia score at the onset of BSIs). It might be that KPC carriage is collinear with severity of illness, and thus mortality might not be necessarily associated with KPC but rather with severity. Cox analysis subsequently showed *bla*_KPC_ was an independent predictor of 14-day mortality, as well as Charlson comorbidity index, Pitt bacteremia score, and septic shock. Thus, KPC-KP BSIs are associated with a poor outcome. The mortality outside the ICUs in present study was 15.3% (33/216), which although significantly lower than that of patients stayed in ICUs (15.3% vs 38.9%, *p* = 0.005) but still relative high. Among the 27 patients stayed in hepato-biliary unit suffering from liver transplantation, 9 patients had fatal outcome at day 14. So, the high mortality of these patients (33.3%) might attribute to the overall high mortality of patients outside the ICUs. Additionally, the *K. pneumoniae* strains isolated from these 9 patients were all positive for *bla*_KPC_, which further confirmed that the KPC-KP BSIs were associated with a poor outcome.

The 14-day mortality of the three cases caused by KPC-producing HMKP was 100% in our study. Zhang et al. reported three cases of KPC-producing HMKP infections, in which two were survived and one unknown [32]. However, there was only one BSIs case. In another study, Zhang et al. reported five cases of KPC-producing HMKP BSIs with 100% mortality [22]. Although limited information available, it seemed KPC-producing HMKP BSIs had disastrous outcome.

## Conclusions

Our study showed the resistance characteristics and clinical manifestation were significantly different between HMKP BSIs and KPC-KP BSIs. HMKP were usually antibiotic susceptible and associated with favorable outcome of BSIs. *bla*_KPC_ was an independent predictor of poor outcome. The emergence of KPC-producing HMKP is a potential threat of public health and must be critically monitored.

## Additional files

Additional file 1: **Table S1.** Non-susceptible rates of 15 antimicrobial agents for 285 *K. pneumoniae* isolates from bloodstream infections according to *bla*_KPC_ and HM phenotype. (DOC 46 kb)

Additional file 2: **Table S2.** Factors associated with hypermucoviscosity phenotype in *K. pneumoniae* isolated from BSIs. (DOCX 16 kb)

Additional file 3: **Figure S1.** Characteristics of 54 *K. pneumoniae* isolates from the non-survival subgroup. (PDF 1596 kb)

## Abbreviations

APACHE: Acute Physiology and Chronic Health Evaluation
BSIs: Bloodstream infections
CI: confidence interval
cKP: classic *K. pneumoniae*
CLSI: Clinical Laboratory Standards Institute
CNS: central nervous system
HM: hypermucoviscous
HMKP: hypermucoviscous *K. pneumoniae*
HR: hazard ratio
hvKP: hypervirulent *K. pneumoniae*
ICU: intensive care units
IQR: interquartile range
KPC: *K. pneumoniae* carbapenemase
KPC-KP: KPC-producing *K. pneumoniae*
MICs: minimum inhibitory concentrations
MLST: multilocus sequence typing
MODS: multiple organ dysfunction syndrome
SD: standard deviation
