# Supplementary material for: Bloodstream infections caused by Klebsiella pneumoniae: prevalence of blaKPC, virulence factors and their impacts on clinical outcome
Source: BMC Infect Dis. 2018 Jul 31;18:358. doi: 10.1186/s12879-018-3263-x (PMC6069789; doi:10.1186/s12879-018-3263-x)
Supplement: Supplementary file 1 — Table S1. Non-susceptible rates of 15 antimicrobial agents for 285 K. pneumoniae isolates from bloodstream infections according to blaKPC and HM phenotype. (DOC 46 kb) [file 12879_2018_3263_MOESM1_ESM.doc]

Table S1. Non-susceptible rates for 285 *K. pneumoniae* isolates from bloodstream infections according to *bla*_KPC_ and HM phenotype.

| **Antimicrobial agents** | **Non-susceptible rates (%)** | | | | | | |
| --- | --- | --- | --- | --- | --- | --- | --- |
|  | **Total**  **(n=285)** | ***bla*_KPC_^+^**  **(n=95)** | ***bla*_KPC_^-^**  **(n=190)** | ***P* Value^a^** | **HMKP**  **(n=69)** | **cKP**  **(n=216)** | ***P* Value^b^** |
| Ampicillin | 100 | 100 | 100 | - | 100 | 100 | - |
| Ampicillin-sulbactam | 55.4 | 98.9 | 33.7 | <0.001 | 15.9 | 68.1 | <0.001 |
| Piperacillin-tazobactam | 38.6 | 96.8 | 9.5 | <0.001 | 4.3 | 49.5 | <0.001 |
| Ceftazidime | 42.8 | 97.9 | 15.3 | <0.001 | 7.2 | 54.2 | <0.001 |
| Cefotriaxone | 45.3 | 96.8 | 19.5 | <0.001 | 7.2 | 57.4 | <0.001 |
| Cefepime | 38.6 | 94.7 | 10.5 | <0.001 | 4.3 | 49.5 | <0.001 |
| Aztreonam | 42.8 | 97.9 | 15.3 | <0.001 | 7.2 | 54.2 | <0.001 |
| Ertapenem | 34.7 | 95.8 | 4.2 | <0.001 | 5.8 | 44 | <0.001 |
| Imipenem | 33.3 | 94.7 | 2.6 | <0.001 | 5.8 | 42.1 | <0.001 |
| Amikacin | 23.9 | 60.0 | 5.8 | <0.001 | 1.4 | 31 | <0.001 |
| Gentamicin | 36.5 | 73.7 | 17.9 | <0.001 | 4.3 | 46.8 | <0.001 |
| Ciprofloxacin | 43.5 | 96.8 | 16.8 | <0.001 | 5.8 | 55.6 | <0.001 |
| Levofloxacin | 40.4 | 94.7 | 13.2 | <0.001 | 4.3 | 51.9 | <0.001 |
| Trimethoprim-sulphamethoxazole | 34.7 | 56.8 | 23.7 | <0.001 | 5.8 | 44 | <0.001 |
| Tigecycline | 1.8 | 4.8 | 1.1 | 0.30 | 0 | 3.3 | 0.58 |

Abbreviations: HM, hypermucoviscous; HMKP, hypermucoviscous *K. pneumoniae*; cKP, classic *K. pneumoniae*.

^a^ *bla*_KPC_^+^ vs *bla*_KPC_^-^.

^b^ HMKP vs cKP.
