# Supplementary material for: Bloodstream infections caused by Klebsiella pneumoniae: prevalence of blaKPC, virulence factors and their impacts on clinical outcome
Source: BMC Infect Dis. 2018 Jul 31;18:358. doi: 10.1186/s12879-018-3263-x (PMC6069789; doi:10.1186/s12879-018-3263-x)
Supplement: Supplementary file 2 — Table S2. Factors associated with hypermucoviscosity phenotype in K. pneumoniae isolated from BSIs. (DOCX 16 kb) [file 12879_2018_3263_MOESM2_ESM.docx]

Table S2. Factors associated with hypermucoviscosity phenotype in *K. pneumoniae* isolated from BSIs

| Variable | No. of isolates  (n=285) | Hypermucoviscosity | |  | Univariate analysis | |  | Multivariate analysis | |
| --- | --- | --- | --- | --- | --- | --- | --- | --- | --- |
|  |  | Positive  (n=69) | Negative  (n=216) |  | OR (95% CI) | *P* |  | OR (95% CI) | *P* |
| K1 | 28 | 18 | 10 |  | 7.3 (3.2-16.7) | <0.001 |  |  |  |
| K2 | 22 | 21 | 1 |  | 94.1 (12.4-716.4) | <0.001 |  | 13.8 (1.8-108.9) | 0.013 |
| *rmpA* | 90 | 62 | 28 |  | 54.5 (24.8-142.9) | <0.001 |  | 40.8 (16.6-100.0) | <0.001 |
| *magA* | 24 | 17 | 7 |  | 9.8 (3.8-24.8) | <0.001 |  |  |  |
| *bla*_KPC_ | 95 | 3 | 92 |  | 0.06 (0.02-0.2) | <0.001 |  |  |  |

Chi-square test or Fisher exact test was used for univariate analysis, and logistic regression for multivariate analysis.
