# Supplementary material for: Bloodstream infections caused by Klebsiella pneumoniae: prevalence of blaKPC, virulence factors and their impacts on clinical outcome
Source: BMC Infect Dis. 2018 Jul 31;18:358. doi: 10.1186/s12879-018-3263-x (PMC6069789; doi:10.1186/s12879-018-3263-x)
Supplement: Supplementary file 3 — Figure S1. Characteristics of 54 K. pneumoniae isolates from the non-survival subgroup. (PDF 1596 kb) [file 12879_2018_3263_MOESM3_ESM.pdf]

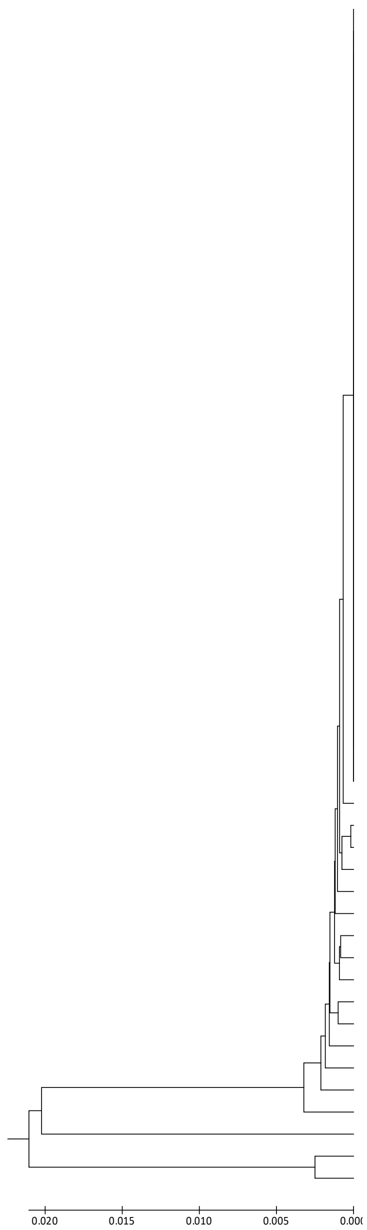

Isolates	Sequence type	*bla* KPC	HM	Serotype	*rmpA*	*magA*	Hostitalization unit	Infection acquisition
1	11	+	-	NT	-	-	Medical	N
2	11	+	+	NT	-	-	ICU	N
4	11	+	-	NT	-	-	Medical	N
5	11	+	-	NT	-	-	Medical	N
6	11	+	-	NT	-	-	Medical	N
7	11	+	-	NT	-	-	Medical	N
8	11	+	-	NT	-	-	Medical	N
11	11	+	-	NT	-	-	ICU	N
12	11	+	-	NT	-	-	Medical	C
15	11	+	-	NT	-	-	Medical	N
16	11	+	-	NT	-	-	Medical	N
18	11	+	-	NT	-	-	Medical	N
19	11	+	-	NT	-	-	Medical	N
20	11	+	-	NT	-	-	Medical	N
21	11	+	-	NT	-	-	Medical	N
22	11	+	-	NT	-	-	ICU	N
25	11	+	-	NT	-	-	ICU	N
26	11	+	-	NT	-	-	ICU	N
27	11	+	-	NT	-	-	Medical	N
28	11	-	-	NT	-	-	Medical	N
30	11	+	-	NT	-	-	ICU	N
32	11	+	-	NT	-	-	Medical	N
33	11	+	-	NT	-	-	Medical	N
34	11	+	-	NT	-	-	Medical	N
36	11	+	-	NT	-	-	ICU	N
37	11	+	-	NT	-	-	ICU	N
39	11	+	-	NT	-	-	ICU	N
40	11	+	-	NT	-	-	ICU	N
41	11	+	-	NT	-	-	ICU	N
42	11	+	-	NT	-	-	ICU	C
45	11	+	-	NT	-	-	Medical	N
46	11	+	-	NT	-	-	Medical	N
48	11	+	-	NT	-	-	Medical	N
49	11	+	-	NT	-	-	Medical	N
50	11	+	-	NT	-	-	Medical	N
54	11	+	-	NT	-	-	ICU	N
23	437	+	-	NT	-	-	ICU	N
9	23	-	+	K1	+	+	Medical	N
38	1660	+	-	K1	+	-	Medical	N
51	152	+	-	NT	-	-	ICU	N
47	2326	-	-	NT	-	-	ICU	N
43	893	+	+	K20	+	-	ICU	N
10	268	-	+	K20	+	-	Medical	N
44	17	-	-	NT	-	-	Medical	C
13	29	-	+	K54	+	-	Medical	N
35	35	+	-	NT	-	-	ICU	N
53	15	+	+	NT	-	-	ICU	C
29	1049	+	-	NT	-	-	Medical	C
17	2328	-	-	NT	-	-	ICU	N
52	364	-	-	NT	-	-	Medical	N
14	2159	-	+	K1	+	+	ICU	C
31	367	-	+	K1	+	+	Medical	N
3	347	-	-	NT	-	-	Medical	N
24	2327	-	-	NT	-	-	Medical	N

Figure S1. Characteristics of 54 *K. pneumoniae* isolates from the non-survival subgroup. The unweighted paired group mean analysis (UPGMA) dendrogram was constructed using the concatenated sequence of MLST. HM, hypermucoviscous; NT, non-typed; C, community; N, nosocomial.
